# Glucocorticoid stimulation increases cardiac contractility by SGK1-dependent SOCE-activation in rat cardiac myocytes

**DOI:** 10.1371/journal.pone.0222341

**Published:** 2019-09-09

**Authors:** Michael Wester, Anton Heller, Michael Gruber, Lars S. Maier, Christian Schach, Stefan Wagner

**Affiliations:** 1 University Heart Center Regensburg, University Hospital Regensburg, Regensburg, Germany; 2 Clinic for Anesthesiology, University Hospital Regensburg, Regensburg, Germany; Rush University Medical Center, UNITED STATES

## Abstract

**Aims:**

Glucocorticoid (GC) stimulation has been shown to increase cardiac contractility by elevated intracellular [Ca] but the sources for Ca entry are unclear. This study aims to determine the role of store-operated Ca entry (SOCE) for GC-mediated inotropy.

**Methods and results:**

Dexamethasone (Dex) pretreatment significantly increased cardiac contractile force *ex vivo* in Langendorff-perfused *Sprague-Dawley* rat hearts (2 mg/kg BW i.p. Dex 24 h prior to experiment). Moreover, Ca transient amplitude as well as fractional shortening were significantly enhanced in Fura-2-loaded isolated rat ventricular myocytes exposed to Dex (1 mg/mL Dex, 24 h). Interestingly, these Dex-dependent effects could be abolished in the presence of SOCE-inhibitors SKF-96356 (SKF, 2 μM) and BTP2 (5 μM). Ca transient kinetics (time to peak, decay time) were not affected by SOCE stimulation. Direct SOCE measurements revealed a negligible magnitude in untreated myocytes but a dramatic increase in SOCE upon Dex-pretreatment. Importantly, the Dex-dependent stimulation of SOCE could be blocked by inhibition of serum and glucocorticoid-regulated kinase 1 (SGK1) using EMD638683 (EMD, 50 μM). Dex preincubation also resulted in increased mRNA expression of proteins involved in SOCE (stromal interaction molecule 2, STIM2, and transient receptor potential cation channels 3/6, TRPC 3/6), which were also prevented in the presence of EMD.

**Conclusion:**

Short-term GC-stimulation with Dex improves cardiac contractility by a SOCE-dependent mechanism, which appears to involve increased SGK1-dependent expression of the SOCE-related proteins. Since Ca transient kinetics were unaffected, SOCE appears to influence Ca cycling more by an integrated response across multiple cardiac cycles but not on a beat-to-beat basis.

## Introduction

Various clinical reports indicate a cardioprotective effect of glucocorticoids (GC) in the context of acute ischemia. GC receptor activation ameliorates biochemical (creatine kinase) and histological myocardial damage as well as improves coronary flow in animals after ischemia/reperfusion [1–3]. In human beings, GC-pretreatment reduces troponin levels and increases cardiac index after cardiopulmonary bypass surgery [4–6]. Besides the well-known anti-inflammatory effects of GC [7], GC-receptor activation has also been shown to stimulate cardiac contractile force in Langendorff-perfused rat hearts [8] and Ca transient amplitude *in vitro/ex vivo* in pig cardiomyocytes after cardiopulmonary bypass surgery [9]. The mechanisms, however, are insufficiently understood. Interestingly, it has been shown that exposure to dexamethasone (Dex) induces store-operated Ca entry (SOCE) in a cellular model of striated muscle cells (L6 myotubes) [10]. In HL-1 cardiomyocytes, stimulation of the serum and glucocorticoid-regulated kinase 1 (SGK1) by Dex (500 nM, 24h) increased expression of the sodium-proton-exchanger (Na^+^/H^+^-exchanger) [11]. In non-cardiac cells, there is evidence that activation of SGK1 may be important for GC-dependent transcriptional regulation of stromal interaction molecule 1 (STIM1) and ORAI calcium release-activated calcium modulator 1 (Orai1) in megakaryocytes [11], HEK293-cells and bone marrow-derived mast cells [12].

The relevance of SOCE for excitation-contraction coupling in healthy adult cardiac myocytes is still controversial [13,14]. SOCE has been shown to be present in neonatal rat cardiac myocytes but hardly detectable in healthy adult cardiac myocytes, and re-emerges during states of increased cardiac stress [14,15]. Growing evidence suggests that pressure-overload (thoracic aortic constriction and abdominal aortic banding) and exposure to phenylephrine or angiotensin II results in stimulation of SOCE and SOC channels, which may be important for development of ventricular hypertrophy by triggering calcineurin-NFAT-signaling in rat cardiomyocytes [16,17].

Since SOCE has been shown to be a major source of Ca entry in non-excitable cells [18], we hypothesized that SOCE, possibly via an SGK1-dependent mechanism, may also contribute to the positive inotropic effect of GC-receptor stimulation in cardiac myocytes.

We show here that *in vivo* exposure of rats to the GC receptor stimulator Dex resulted in increased systolic contractile function (isolated Langendorff-perfused hearts). Moreover, we show that 24h *in situ* exposure of isolated rat ventricular myocytes to Dex increased Ca transient amplitude and sarco/endoplasmic reticulum (SR) Ca content by a SGK1-SOCE-dependent fashion.

## Methods

### Animals

Male *Sprague-Dawley* rats (Charles River, Europe) were purchased at the age of 12 weeks. The animals were housed on a 12/12 h light/dark cycle with constant temperature (22–23°C) with access to food and tap water ad libitum. At the age of 20 weeks, experiments were performed. For some experiments, Dex (2 mg/kg BW) vs. vehicle was injected i.p. 24 h before. Euthanasia was performed using decapitation after isofluran (2%) anesthesia. All animal care and experimental procedures followed German law as well as the Guide for the Care and Use of Laboratory Animals, published by the National Institutes of Health, and were approved by the Institutional Review Board at the University of Regensburg and by the district government of Lower Franconia (55.2-2532-2). All efforts were made to minimize suffering.

### Langendorff-perfused hearts

After isofluran (2%) anesthesia, hearts were isolated and the aorta was rapidly cannulated. During preparation hearts were continuously retrogradely perfused with a cold (6.2+/−0.2°C), oxygenated, modified Krebs-Ringer solution and transferred to a Langendorff apparatus (Hugo Sachs Electronic KG, March-Hugstetten, Germany). The modified Krebs-Ringer’s salt solution, containing in mM: NaCl 124.5, KCl 4.5, CaCl_2_ 2.5, KH_2_PO_4_ 1.2, NaHCO_3_ 15.5, EDTA 0.05, glucose 11.5, pyruvate 2, mannitol 10, and insulin 5 units per liter) was filtered in-line (5 μm pore size filter disk, Sigma-Aldrich, Munich, Germany). Krebs-Ringer solution was equilibrated with 95% oxygen and 5% carbon dioxide. A fluid-filled balloon was introduced into the left ventricle. Left ventricular end diastolic pressure (LVEDP) was kept at 0 mmHg during the stabilization period. Hearts were perfused with a constant pressure of 55 mmHg. After 30 minutes of equilibration, measurements were performed. For the Langendorff experiments, all parameters were derived from the pressure signal. Time to peak was analyzed using the LabChart Pro (version 8.1.9) peak analysis tool. The tool acquired each parameter (developed LVP, time to peak, dp/dt, etc.) from an average of 20 contraction cycles. The starting point for the rising phase was defined as the event when the pressure value left the baseline band of sample values. The baseline band was defined as the sequence of points where the variation was less than 5% of the developed pressure amplitude in either direction. The peak was identified as the point of maximum (positive deflection) of the averaged pressure signal if an absolute increase in pressure exceeded 30 mmHg (threshold). The time to peak was calculated as the difference between the times at starting point and at peak.

### Cardiomyocyte isolation and culture

Hearts were quickly removed from anesthetized (2% isoflurane) rats at the age of 20 weeks (body weight: 250–350 g) and transferred to ice cold buffer solution (containing in mM: NaCl 113, KCl 4.7, KH_2_PO_4_ 0.6, Na_2_HPO_4_ 0.6, MgSO_4_ 1.2, NaHCO_3_ 10, KHCO_3_ 12, Hepes 10, BDM 0.05, Glucose 5.5; pH adjusted to 7.4 at 37°C). The aorta was identified, connected to a cannula and rinsed to wash out remaining blood, proven by decoloration of the cardiac tissue. Thereafter, the heart was mounted on a Langendorff apparatus (Hugo Sachs Electronic KG, March-Hugstetten, Germany) and the heart was perfused retrogradely with prewarmed enzyme solution (trypsin 0.6%, 7.5 mg/mL liberase, Roche, 37°C) for 15 minutes. Atria and the right ventricle were removed and the left ventricle was cut into small cubicles and carefully triturated with a Pasteur pipette. Cells were allowed to settle under gravity for 10 minutes in buffer solution (containing in mM: NaCl 113, KCl 4.7, KH_2_PO_4_ 0.6, Na_2_HPO_4_ 0.6, MgSO_4_ 1.2, NaHCO_3_ 10, KHCO_3_ 12, Hepes 10, BDM 0.05, Glucose 5.5; pH adjusted to 7.4 at 37°C). After stepwise increase of Ca (0.2, 0.4, 0.8 mM), the cell suspension was transferred to cell culture medium (MEM with addition of insulin-transferrin-selenium, L-glutamine, penicillin, streptokinase, 2 mM BDM, BSA) and incubated with Dex (0.010 mg/mL) vs. vehicle for 24 h (37°C, 20% O_2_, 75% N_2_, 5% CO_2_). In some experiments the SGK-1-inhibitor EMD (50 μM) was added.

### Measurement of intracellular Ca concentration and sarcomere length acquisition

Ca measurements were conducted as previously described. [19] Briefly, myocytes were plated on laminin coated glass chambers and allowed to attach for 20 minutes. Thereafter, myocytes were loaded with FURA-2-AM (5 μM; 0.02% w/v Pluronic; 10 minutes, 25°C) and the chambers were mounted on stage of an epifluorescence microscope (IonOptix). After washing out the external dye (10 min) by superfusion with modified Tyrode’s solution (containing in mM: NaCl 140, KCl 4, MgCl_2_ 1, Hepes 5; pH adjusted to 7.4, at 37°C), cell twitching was elicited by electrical field stimulation (20 V, 1 Hz). For Ca measurements, FURA-2 was alternating excited at wavelengths of 340 nm and 380 nm (240 Hz) and emitted fluorescence acquired by a photomultiplier at 510 nm (all optical filters by Chroma Technology Corporation, wavelength ±10 nm). For each individual experiment, unspecific background fluorescence was measured at each wavelength in immediate vicinity of each cell and subtracted from the fluorescence intensities emitted from the cell. The magnitude of the background fluorescence was less than 10% of the total cell fluorescence. Ca transient properties were calculated from a mean of 10 electrically stimulated transients. Time to peak was measured from the onset of the Ca transient, not from the pacing event marker, to the peak of the transient. In parallel to fluorescence acquisition, sarcomere length was measured as a function of time by means of a video camera system. Fractional shortening was calculated as absolute sarcomere shortening normalized to diastolic sarcomere length (IonWizard). For some experiments, sarcomere shortening was recorded in absence of FURA-2 to avoid Ca buffering effects. For some experiments, myocytes were exposed to SKF-96356 (SKF, 2 μM) or BTP2 (5 μM) to inhibit SOCE. Caffeine (10 mM) was administered to measure SR content at the end of each experiment

### SOCE measurements

A specific protocol was used to measure SOCE. SR Ca was depleted by caffeine (10 mM) after electrical stimulation until steady state (electrical field stimulation; 1 Hz; 20 V). Then, the superfusion with modified Tyrode’s solution (containing in mM: NaCl 140, KCl 4, MgCl_2_ 1, Hepes 5; pH adjusted to 7.4) was switched to 0 Ca/0 Na solution (containing in mM: LiCl 140, KCl 4, MgCl_2_ 1, Hepes 5, EGTA 1) and myocytes were incubated with thapsigargin (Tg, 100 nM) and verapamil (10 μM) for 5 minutes. Repeated administration of caffeine (10 mM) ensured maximal depletion of SR Ca stores. Thereafter, extracellular Ca concentration was raised to 2.5 mM and intracellular Ca concentration was measured continuously as described. For some experiments, 2 μM SKF was added 5 minutes before and during elevation of extracellular Ca.

### mRNA measurements

After cell culture in the presence of Dex (1 mg/mL) or EMD (50 μM) or both for 24 h, cells were harvested and frozen at -80°C until further processing (RNeasy Mini Kit, Qiagen, Netherlands). Total mRNA was extracted for STIM1/2, Orai1/2/3 and TRPC channels 1/3/4/6 (transient receptor potential cation channels) and contents were assessed by quantitative reverse-transcriptase polymerase chain reaction and normalized with β-Actin (ViiA7 Life Technologies, Applied Biosystems). Primers are shown in S1 Table. Content of mRNA was calculated relative to β-Actin using the standard curve-method.

### Data analysis and statistics

Statistical analysis was performed using Graphpad Prism (Version 6.01 for Windows, GraphPad Software, La Jolla California USA). Data are presented as mean ± standard error of the mean (SEM). After testing for normal distribution using the D’Agostino-Pearson-test and the Kolmogorov-Smirnov-test, data were compared using either a paired t-test or one-way ANOVA. Values of p<0.05 were considered significant. SOCE curves were analyzed by exponential curve fitting with Graphpad Prism (two-phase association, least squares ordinary fit). The computed rate constant K_fast_ was used as a measurement for the velocity of SOCE.

## Results

### Positive inotropic effect of dexamethasone

**Fig 1:**
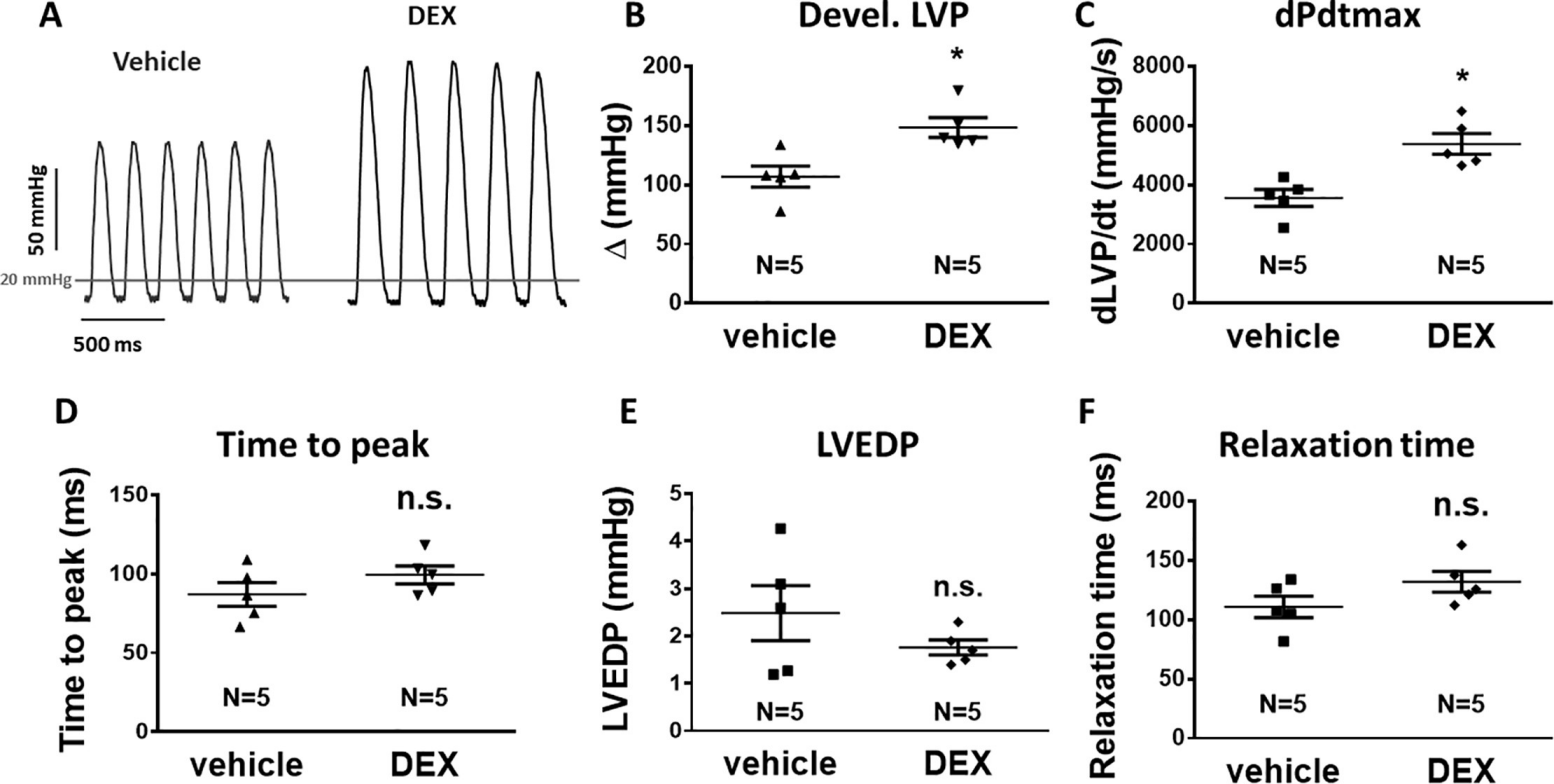
Dex significantly increased inotropy in Langendorff-perfused rat hearts. (A) Exemplary original tracings of left ventricular pressure (LVP) as a function of time of spontaneously beating Langendorff-perfused rat hearts. Mean data for systolic developed pressure (devel. LVP, B) and maximal pressure development rate (dPdt_max_), (C). 24 h Dex pre-treatment (i.p., 2 mg/kg BW) significantly increased devel. LVP and dPdt_max_ consistent with a strong positive inotropic effect. (D) Mean data for total time to peak systolic LVP (time to peak). In contrast to systolic function, diastolic function was not affected by Dex pretreatment as indicated by mean data for left ventricular end-diastolic pressure (LVEDP, E) and relaxation time (F). *—p<0.05 vs. vehicle (unpaired t-test, n = 5 hearts in each group).

First, we examined the effects of Dex on cardiac contractility in a Langendorff-perfused whole heart setup. Original tracings of left ventricular pressure as a function of time are shown in Fig 1A. *In vivo* Dex-pretreatment (2 mg/kg BW i.p., 24h prior to experiment) resulted in a significant increase in developed left ventricular (systolic) pressure (developed LVP, LVP amplitude) as well as maximal pressure development rate (dPdt_max_, Fig 1A–1C). The LVP amplitude increased from 107±8.9 to 148±8.3 mmHg (N = 5 vs. 5, p = 0.0093, Fig 1B), and dPdt_max_ increased from 3356±284 to 5379±349 mmHg/s (N = 5 vs. 5, P = 0.0037, Fig 1C). Interestingly, the time to peak contraction, which is dependent on the coupled gating of L-type Ca channels (LTCC) and ryanodine receptor (RyR) Ca-induced Ca release (CICR) [20], was not affected by Dex pretreatment (Fig 1D) suggesting that the Dex-dependent positive inotropic effect does not involve stimulation of LTCC function. In addition, Dex-pretreatment did not alter diastolic contractile function (Fig 1E and 1F). After Dex pretreatment, both left ventricular end-diastolic pressure (LVEDP, Fig 1E) and relaxation time (Fig 1F) were not different from vehicle. The latter suggest that diastolic Ca removal may also not be altered by Dex pretreatment.

### Dexamethasone increased intracellular Ca transient amplitude by a SOCE-dependent mechanism

**Fig 2:**
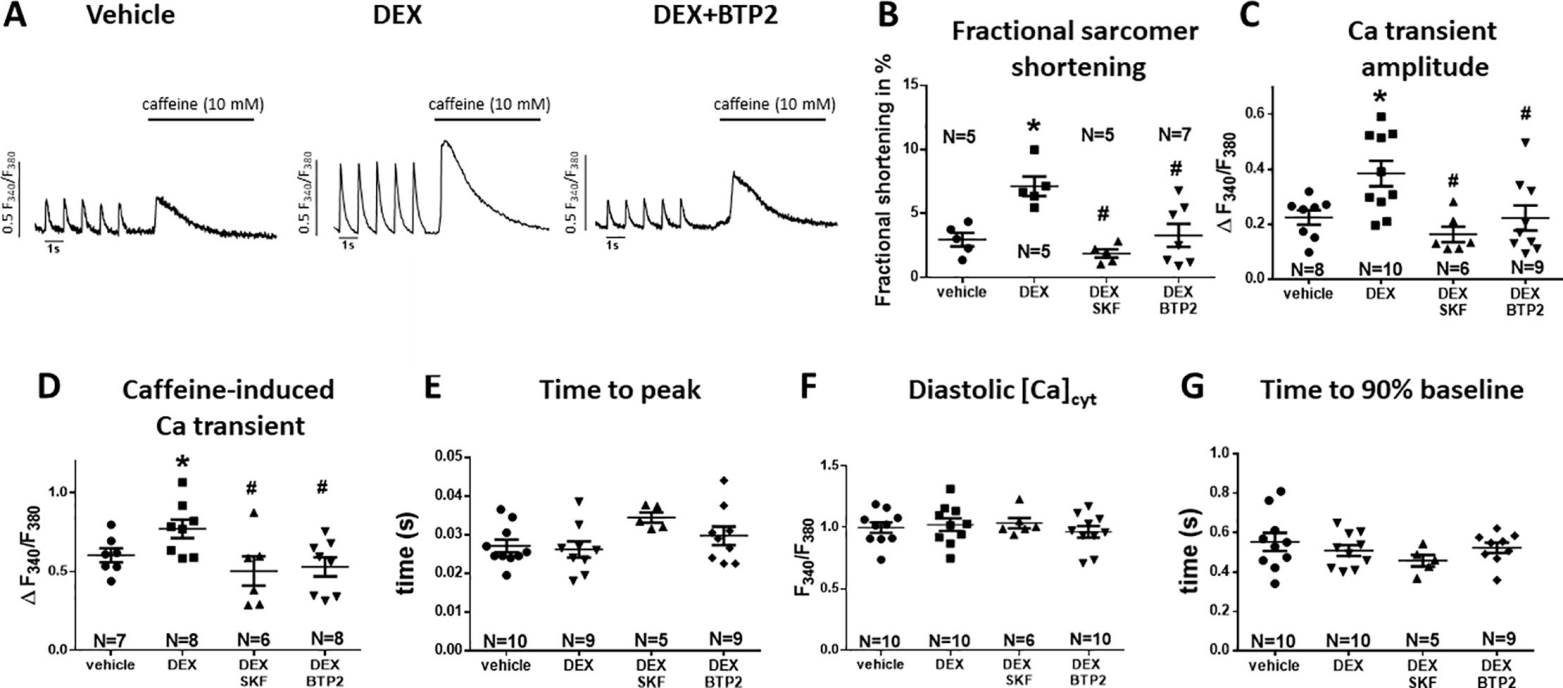
Dex increased Ca transient amplitude by a SOCE-dependent mechanism. (A) exemplary original tracings of electrical field-stimulated Ca transients (1 Hz, 20 V; marked by ticks) and caffeine-transients (10 mM) of FURA-2 loaded isolated ventricular myocytes cultured for 24 h with either vehicle, Dex or Dex in the presence of SOCE inhibitor BTP2. Mean data for fractional shortening (B), Ca transient amplitude (D), Caffeine-transient amplitude (D), time to peak Ca transient (peak time, E), diastolic Ca (F), and time to 90% relaxation of the Ca transient (G) are also shown. 24 h Dex-pretreatment (1 mg/mL) significantly increased fractional shortening, Ca transient amplitude, and caffeine-induced Ca transient. This could be prevented by addition of SOCE inhibitors SKF (2 μM) or BTP2 (5 μM). Peak time, diastolic Ca, and time to 90% baseline were unaltered in all groups. n = 5–10 animals for each group. *P<0.05 vs vehicle, #P<0.05 vs Dex (one-way ANOVA).

To further investigate the mechanism of the Dex-dependent increase in systolic contractility, we measured intracellular Ca handling in FURA-2 loaded isolated rat ventricular myocytes (Fig 2). Consistent with an increase in cellular shortening (fractional shortening), 24 h Dex-pretreatment significantly increased Ca transient amplitude (Fig 2A and 2C). Interestingly, this increase was accompanied by a significant increase in SR Ca load as assessed by rapid exposure to caffeine (10 mM, Fig 2A and 2D). Compared to vehicle, caffeine-transient amplitude increased from 0.62±0.12 to 0.77±0.17 (for vehicle vs. Dex, N = 7 vs. 8, p = 0.0036) suggesting an SR-dependent mechanism of the positive inotropic effect. In accordance with unaltered time to peak LVP (Fig 1), the time to peak Ca transient was not affected by Dex pretreatment (Fig 2E) supporting the hypothesis that stimulation of LTCC is not crucially involved in the Dex-dependent positive inotropic effect. Moreover, Dex exposure did not affect fractional SR Ca release (S1A Fig). Fractional release changes in parallel with LTCC for a given SR Ca load. In the face of increased SR Ca load, a Dex-dependent increased in LTCC is, thus, highly unlikely and cannot be responsible for the positive inotropic effect of Dex.

In addition, diastolic Ca concentration and time to 90% relaxation of the Ca transient, which indicate function of SR Ca ATPase (SERCA) [20], were not different from vehicle (Fig 2F and 2G) suggesting that stimulation of SR Ca reuptake may also not underlie the Dex-dependent increase in Ca transient amplitude. Finally, we did not detect any difference in caffeine-transient decay rate as a measure of NCX function (S1B Fig).

To test, if Ca entry by a store-related mechanism may contribute to the increased Ca transient amplitude upon Dex pretreatment, we exposed myocytes to the selective SOCE inhibitors SKF (2 μM) or BTP2 (5 μM), respectively. Both drugs abolished the Dex-dependent increase in Ca transient amplitude and caffeine-transient amplitude (Fig 2C and 2D). There was a strong and significant correlation between SR Ca content and Ca transient amplitude in all groups (S1C-S1F Fig). Thus, increased SOCE-dependent Ca entry, which fills up the SR Ca store may likely underlie the Dex-dependent increase in Ca transient amplitude.

### Dexamethasone increases SOCE by a SGK1-dependent mechanism

**Fig 3:**
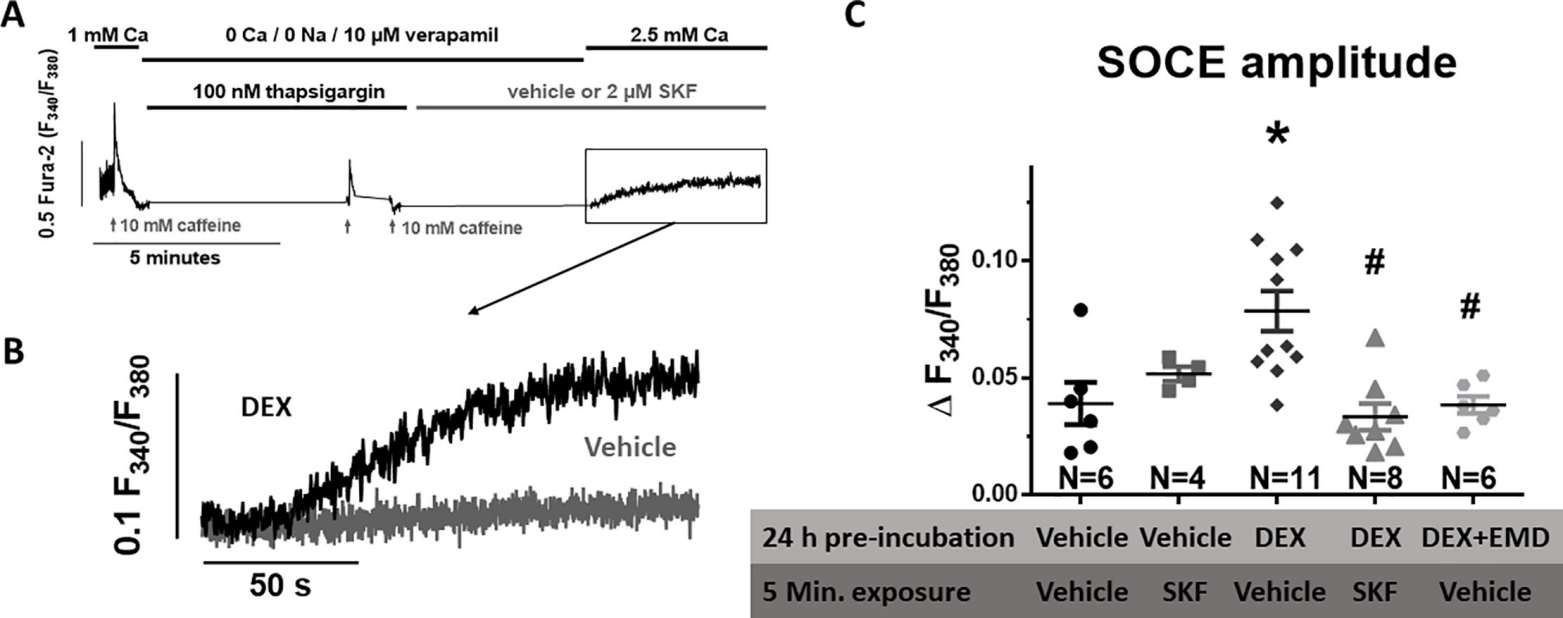
Dex stimulates SOCE via an SGK1-dependent mechanism. (A) Schematic representation of the SOCE-protocol. Isolated rat ventricular myocytes loaded with FURA2 were electrical-field stimulated (1 Hz, 20V) in the presence of 1 mM extracellular Ca concentration. After addition of 10 mM caffeine, the superfusion was changed to Ca/Na-free Tyrode's solution to empty the intracellular Ca stores. The SERCA-inhibitor thapsigargin (100 nM) and LTCC-inhibitor verapamil (10 μM) were added to prevent Ca reuptake into the SR and Ca entry via LTCC and caffeine (10 mM) was repeatedly administered to completely empty the SR. SOCE was then measured after wash in of Ca containing Tyrode. In parallel experiments, SKF (5 min., 2 μM) was added to inhibit SOCE. (B) Original traces of SOCE measured at the end of the protocol in myocytes pre-treated with either vehicle or Dex ((1 mg/mL, 24 h). (C) Mean data for SOCE amplitude. Dex exposure significantly increased SOCE amplitude. Interestingly, exposure to selective the SGK1 inhibitor EMD (24 h, 50 μM) completely abolished the Dex-dependent stimulation of SOCE amplitude. Data are from n = 4–11 animals. *P<0.05 vs vehicle, #P<0.05 vs Dex (one-way ANOVA).

To examine, if Dex pretreatment stimulates SOCE in cardiac myocytes, we used a specific protocol to measure SOCE as detailed in Fig 3A. Dex pretreatment resulted in a significant increase in SOCE (Fig 3B and 3C). Compared to vehicle, SOCE amplitude increased from 0.039±0.22 to 0.079±0.028 (for vehicle vs. Dex, N = 6 vs. 11, p = 0.0003). This increase was abolished in the presence of the SOCE inhibitor SKF (2 μM, Fig 3C). To investigate, if SGK1, which has been shown to be activated by GC stimulation, may be involved in the Dex-dependent regulation of SOCE, we exposed myocytes to the selective SGK1 inhibitor EMD (50 μM). Importantly, EMD exposure completely prevented the Dex-dependent increase in SOCE amplitude (Fig 3C) suggesting that Dex may requires SGK1 to stimulate SOCE.

### Dex pre-treatment enhanced expression of STIM2, TRPC channel 3 and 6 by a SGK1-dependent mechanism

**Fig 4:**
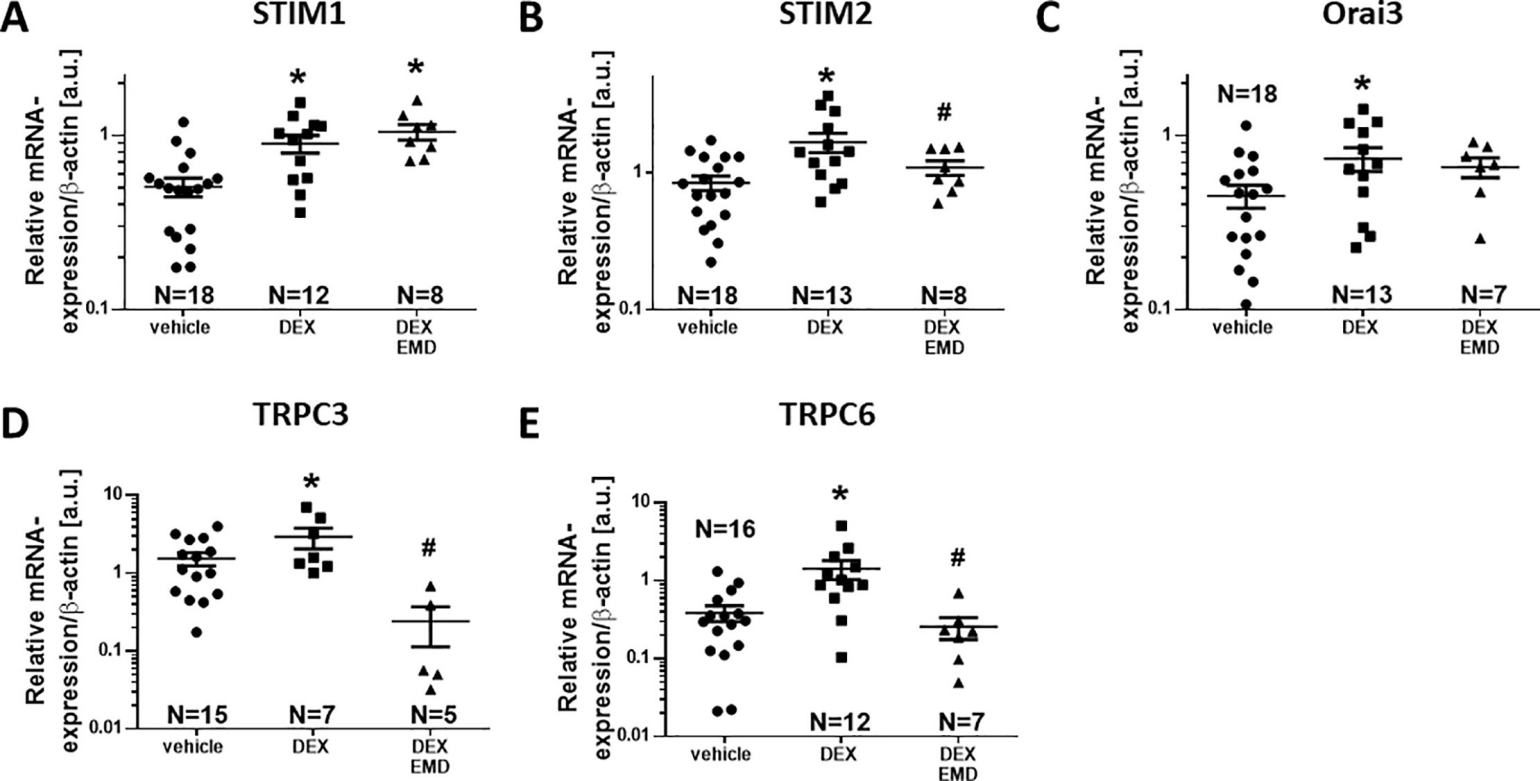
Dex incubation increases m-RNA expression of SOCE-proteins in cardiac myocytes. Mean data for mRNA levels measured via real-time PCR deploying the standard-curve method are shown as relative expression versus β-actin in arbitrary units (a.u.). Dex significantly increased STIM1 (A), STIM2 (B), Orai3 (C), and TRPC3 (D) and TRPC6 (E) expression. Importantly, Dex-dependent stimulation of STIM2, TRPC3 and TRPC6 expression were prevented by EMD pretreatment (B, D, and E, respectively). Data are from n = 5–18 animals. *P<0.05 vs vehicle, #P<0.05 vs Dex (one-way ANOVA).

To test, if the Dex-dependent stimulation of SOCE may result from increased cardiac expression of SOCE-related proteins, we measured mRNA levels of STIM (1, 2), Orai (1–3) and TRPC (1,3,4,6) by quantitative PCR. Intriguingly, compared to vehicle, 24 h Dex pre-treatment significantly increased the mRNA expression of STIM 1 and 2 (by 76.9±20.8% and 98.2±31.8%, respectively, p = 0.0012 and p = 0.0014 respectively, N = 12 and 13) as well as TRPC channels 3 and 6 (by 90.1±57.2% and 268.1±100.4%, respectively, p = 0.0392 and p = 0.0353, respectively, N = 7 and 12) in isolated myocytes (Fig 4, S2 Table). In contrast, mRNA expression of Orai1 was significantly reduced (by 76.8±7.1%, S2 Table). Importantly, the Dex-dependent stimulation of mRNA expression of STIM2, TRPC 3, 6 and Orai1 were abolished by inhibition of SGK1 with EMD (50 μM).

## Discussion

**Fig 5:**
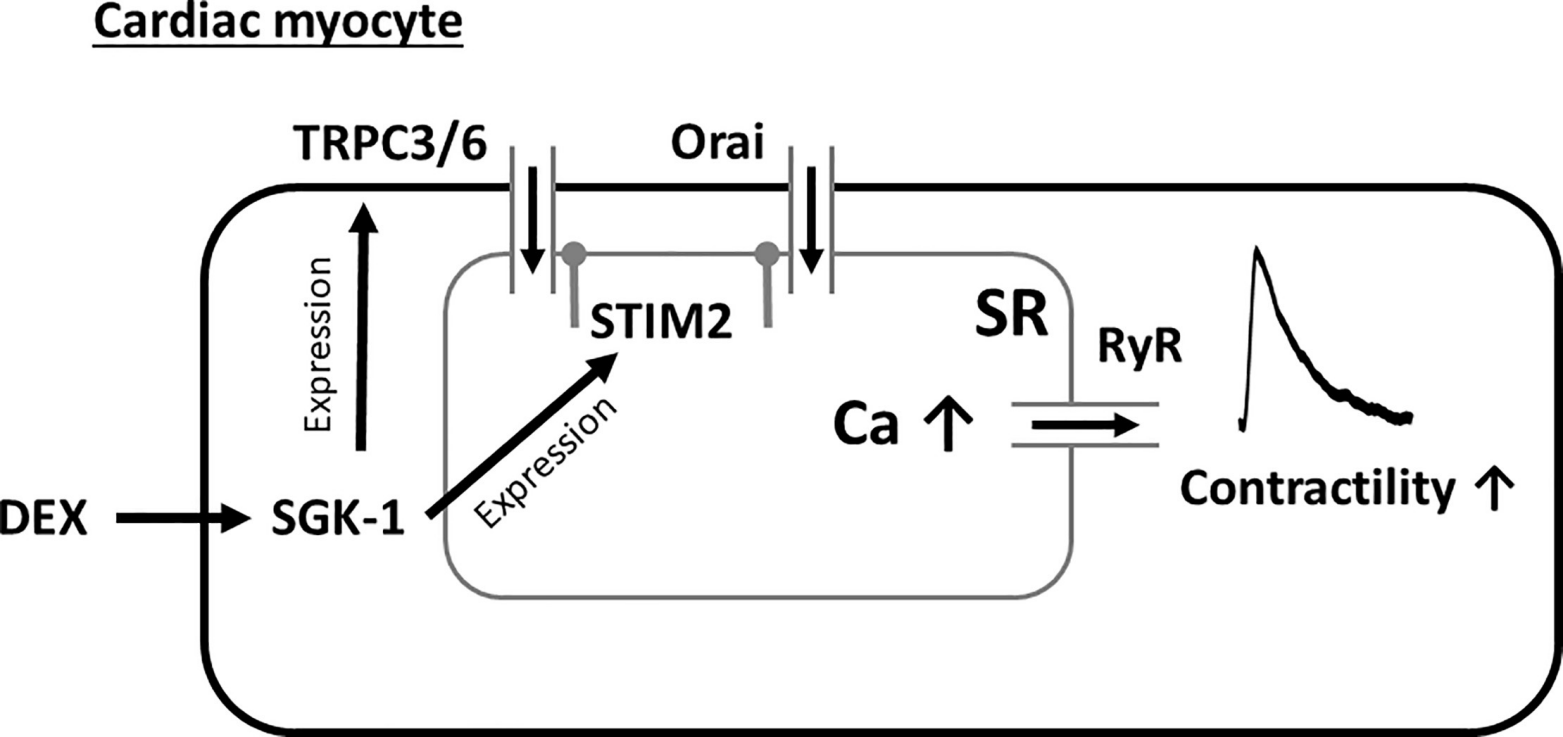
Schematic presentation of the positive inotropic effect of dexamethasone. Dex stimulates mRNA expression of SOC channels TRPC channel 3 and 6 and STIM2 via an SGK1-dependent mechanism. This results in increased SOCE mediated through Orai and TRPC channels leading to an elevation of SR Ca load and improved systolic RyR Ca release, which increases Ca transient amplitude and contractile force.

We show here that exposure to the GC receptor stimulator Dex resulted in increased systolic contractile function of rats *in vivo* (isolated Langendorff-perfused hearts). Moreover, 24 h *in situ* exposure of isolated rat ventricular myocytes to Dex increased Ca transient amplitude and SR Ca content by a SGK1-SOCE-dependent mechanism. In accordance, Dex-pretreatment increased mRNA expression of STIM1, STIM2, Orai3 and TRPC channels 3 and 6 in rat cardiac myocytes. A summary of the proposed mechanisms of inotropy by Dex exposure is shown in Fig 5.

### Exposure to Dex improves cardiac contractile force by a SOCE-dependent mechanism

We show here that *in vivo* Dex-pretreatment (24 h, 2 mg/kg BW) increased developed LVP and dPdtmax in Langendorff-perfused rat hearts (Fig 1A–1C) and increased fractional sarcomere shortening and Ca transient amplitude in isolated cardiomyocytes (24 h incubation *ex vivo*, 0.010 mg/mL) (Fig 2A–2C).

GC have been shown to exert actions through binding to the GC receptor [21] and numerous studies have suggested a positive inotropic effect. Conditional overexpression of human glucocorticoid receptor in mice, for instance, has been shown to increase Ca transient amplitude, cell shortening, and SR Ca load in isolated myocytes compared to control [22]. Similar results have been acquired by GC-receptor stimulation with Dex in adult rats *in vivo* (1 mg/kg BW/day for 5 days) showing increased cardiac contractile force (Langendorff-perfused hearts) [8]. In accordance, exposure to GC-receptor activator methylprednisolone (30 mg/kg BW) 6 h prior to cardiopulmonary bypass surgery has also been shown to increase Ca transient amplitude *in vitro/ex vivo* in pig cardiomyocytes [9].

However, the mechanisms that lead to GC-receptor dependent stimulation of inotropy are less clear. The effect of glucocorticoids on intracellular Ca stores, for instance, has not been systemically examined in cardiomyocytes. Beside data that conditional GC-receptor overexpression may increase SR Ca load in isolated myocytes [22], there is few evidence in myocytes. In non-excitable cultured lymphoblastoid cell lines, however, cortisol incubation (200 nM, 48 h) has been shown to increase SR Ca content [23]. We show here that Dex exposure increased SR content measured by caffeine-induced transients in isolated cardiomyocytes (Fig 2D), which may at least partly explain the Dex-dependent stimulation of inotropy. We have also shown that fractional SR Ca release, which changes in parallel with LTCC for a given SR Ca load, was not different upon DEX exposure (S1A Fig). Since SR Ca load was increased, a Dex-dependent increased in LTCC cannot be responsible for the positive inotropic effect of Dex. Moreover, we show here that was a strong correlation between SR Ca load and Ca transient amplitude for all experimental groups (S1C-S1F Fig) suggesting that increased SR Ca load may be the major mechanism underlying the observed increase in Ca transient amplitude. This raises the question about the source of Ca that leads to increased SR Ca content.

The existence of SOCE and its vital contribution to the physiologic function of many cell types has been extensively studied in non-excitable cells (e.g. immune cells [24]) as well as excitable cells (e.g. skeletal muscle cells [25]). SOCE has been shown to be critically involved in cardiomyocyte maturation and function (for review see [14,15,26]). Multiple reports have also indicated a crucial role of SOCE in the development of cardiac hypertrophy. For instance, increased STIM1-expression and SOCE have been shown to result from exposure of cultured mouse cardiomyocytes to the pro-hypertrophic stimuli angiotensin II or phenylephrine and cellular hypertrophy was prevented by STIM1 knock-down [16,17]. Moreover, trans-aortic banding in mice, which also results in cardiac hypertrophy, was shown to increase STIM1-expression and SOCE [16,17]. In accordance, STIM1 knock-down was shown to prevent cardiac hypertrophy in adult rats subjected to aortic banding [17]. However, a lack of SOCE may also be detrimental. For instance, it was shown that cardio-specific knock-down of STIM1 in mice results in phenotypic dilatative cardiomyopathy [27,28]. Our data underscore the importance of SOCE in the heart by extending its role to regulation of excitation-contraction coupling.

The role of SOCE for excitation-contraction coupling in cardiac myocytes, however, remains unclear and disputed [16,17]. In accordance with previous data [16,29,30], we have measured negligible SOCE in isolated rat cardiac myocytes under basal conditions suggesting that SOCE is not important for basal regulation of excitation-contraction coupling. In contrast, we show here that SOCE amplitude increased dramatically upon GC stimulation with Dex in isolated rat cardiomyocytes. Moreover, the Dex-dependent stimulation of Ca transient amplitude, fractional sarcomere shortening, and caffeine-induced Ca transient were abolished when SOCE inhibitors SKF (2 μM) and BTP2 (5 μM) were present (Fig 2A–2D) suggesting that this Dex-dependent SOCE stimulation appears to be critically involved in the positive inotropic effect. Sedova et al. elegantly showed that SOCE entry rate is graded with SR Ca store depletion in isolated calf pulmonary artery endothelial cells [31]. Given that the same molecular SOCE components are present in skeletal and cardiac myocytes, it is reasonable to assume that a similar graded response may be present in cardiac myocytes. However, since the kinetics of STIM1 redistribution and Orai activation are in the order of seconds not milliseconds [30], it is unlikely that the graded response would be important for beat-to-beat regulation of SR Ca content. Instead, it is more likely that the SOCE response integrates gradual beat-to-beat changes in SR Ca content across many cardiac cycles. Although SOCE may not be relevant during basal cardiac conditions as shown by Luo et al. [16], an intervention that depletes SR Ca stores, could result in SOCE stimulation and SR Ca refilling providing a kind of autoregulation of SR Ca content. However, this graded response across cardiac cycles and for different levels of SR Ca load remains to be shown.

SKF has been widely used to block SOCE. However, there is evidence that SKF may also block LTCC but at a much larger concentration. The IC_50_ for SOCE inhibition is 12 μM in T-lymphocytes [32]. The IC_50_ for LTCC inhibition appears to be around 20–30 μM in smooth muscle cells [33]. In contrast, in neonatal rat ventricular myocytes 10 μM SKF has been shown to have no effect on LTCC function [34]. We have used an even lower concentration (2 μM) rendering inhibition of LTCC function very unlikely. Nevertheless, to verify our results, we have also used the highly-specific SOCE-inhibitor BTP2 [32,35], that showed a similar inhibition of Dex-dependent inotropy as SKF. The IC_50_ of BTP2 for SOCE-inhibition has been determined between 10–150 nM [32]. The range of concentrations commonly used to selectively block SOCE in isolated murine and cat cardiomyocytes varies between 1 μM and 20 μM [36–38]. To date, there is no data that BTP2 may also block LTCC in cardiac myocytes. Thus, is seems plausible that at least part of the Dex-dependent increase in SR Ca content may be mediated by stimulation of SOCE and not be dependent on LTCC. More indirect evidence that LTCC stimulation may not be involved results from our analysis of kinetics of the contraction and Ca transient. We show here that the time to peak contraction, which is known to be dependent on the coupled gating of LTCC and RyR Ca release [20], was not affected by Dex pretreatment (Fig 1D), which is also in accordance to previous data [39]. Obejero et al. found no effect of Dex-pretreatment (200 nM, 48h) on LTCC currents in A7r5 smooth muscle cells [40]. On the other hand, it was shown that prolonged exposure to Dex for 5 days *in vivo* resulted in increased LTCC density in mice and mRNA levels in rats, respectively [40,41]. So, the differences to our findings may be attributed to the much longer Dex-treatment duration (5 days vs 24 h).

In addition to LTCC and RyR, we provide indirect evidence that stimulation of SERCA may also not be the major mechanism of Dex-dependent inotropy. We show here that Dex-pretreatment did not alter diastolic contractile function (Fig 1E and 1F). After Dex pretreatment, both LVEDP (Fig 1E) and relaxation time (Fig 1F) were not different from vehicle. Moreover, the diastolic Ca concentration and time to 90% relaxation of the Ca transient, which indicate function of SERCA [20], were also not different from vehicle (Fig 2F and 2G). This suggests that Dex may not stimulate SR Ca reuptake. However, conclusions about regulation of SERCA function by GC stimulation should be made carefully since our data is in contrast to a previous publications showing an enhanced Ca transient decay by conditional GC-receptor overexpression in mice [22]. On the other hand, prolonged *in vivo* exposure to Dex (oral; 0.35 mg/kg BW; 15 days) has been shown to reduce SERCA expression levels in rats [41]. Another important aspect is the question, why the steady-state autoregulation of Ca fluxes described by the Eisner group, was not able to prevent the increase in Ca transient amplitude upon Dex exposure [42]. If autoregulation would be present, the increased SR Ca load and release (due to increased SOCE) would be accompanied by a reduced LTCC influx due to LTCC inhibition by dyadic Ca. This would result in a stable, unaltered Ca transient amplitude but with increased fractional release. In contrast to this concept, we have measured increased Ca transient amplitude but unaltered time to peak, and no significant differences in fractional Ca release (Fig 2C and 2E; S1A Fig). To get more insight, we have also analyzed the caffeine-transient decay rate as a measure of NCX function (S1B Fig). There was no significant difference in caffeine-transient decay rates between our experimental groups suggesting that NCX function was neither enhanced nor reduced (S1B Fig). This is in accordance with the concept of balanced Ca fluxes. At steady state, the amount of Ca that enters the cell from extracellular space equals the amount of Ca that is removed by NCX [20]. Thus, despite increased SR Ca load and release, the extracellular Ca influx was not reduced in our experimental model. The Eisner concept of autoregulation only works for modest changes. If one parameter is changed too drastically, a new equilibrium will evolve. In our case, Ca transient amplitude increases in parallel to SR Ca load to a new level. The question remains, why the mechanisms of autoregulation described by Eisner are not able to reduce steady-state Ca influx in our model despite increased SR Ca release into thy dyadic cleft. Although there are many possible explanations, it seems plausible that the Ca influx mediated by SOCE, in contrast to LTCC, may not be subject to autoregulation because Ca may be transferred directly from extracellular space into the SR bypassing the dyadic cleft. It is generally assumed that STIM-mediated Orai-activation allows for direct Ca entry into the SR [13,14]. Orai is a trans-sarcolemmal Ca channel in close vicinity to the SR membrane. However, the detailed route, through which Ca enters the SR via SOCE needs to be further elucidated.

Nevertheless, it seems obvious that the Dex-dependent inotropy can be differentiated from the acute inotropic effect secondary to stimulation of the β-adrenergic cascade or stimulation of G_q_ protein-coupled receptors. A stimulation of β-adrenergic receptors would activate protein kinase A and would result in increased phosphorylation of cardiac troponin I, LTCC, phospholamban and RyR [43]. In consequence, trans-sarcolemmal Ca influx and SR Ca reuptake would increase and Ca sensitivity of troponin would be augmented leading to enhanced Ca transient kinetics [20]. The stimulation of G_q_ protein-coupled receptors would also result in increased LTCC-dependent Ca influx, which should increase the time to peak of the Ca transient [20,44]. In contrast to these effects, we have found no significant differences in Ca transient kinetics. Thus, it seems unlikely that our observed changes in Ca transient amplitude and contractility are due to activation of either β-adrenergic or G_q_ protein-coupled receptor-related pathways.

### Dex improves cardiac contractile force via SGK1-dependent upregulation of SOCE-machinery

To investigate the mechanism of Dex-dependent stimulation of SOCE, we measured expression of important proteins involved in SOCE. We show here that *in vivo* Dex-pretreatment (24 h, 2 mg/kg BW) increased mRNA-levels of STIM1, STIM2, Orai3 and TRPC channels 3 and 6 (Fig 4A–4E). To our knowledge, this is the first report directly linking GC receptor stimulation to increased expression of SOC channels in cardiomyocytes. It has to be noted that the relationship between mRNA content and final expression of functional SOCE proteins is not necessarily firm. For STIM1 and Orai1, there is evidence for a correlation between mRNA and protein expression [45]. Therefore, despite the lacking explicit evidence, we believe it would be reasonable to assume that the effects of DEX-incubation and SGK1-inhibition on mRNA levels of SOCE components would translate into parallel changes in SOCE protein expression. The mechanisms linking GC receptor stimulation to increased SOCE protein expression are unclear but may involve gene regulation via SGK1. Dex exposure has been shown to activate SGK1 possibly by phosphorylation via PI3-kinase/ PIP3-dependent kinase PDK1 [46]. As a result, SGK1 facilitates nuclear translocation of NF-κB, which has been shown to induce expression of the SOC channels STIM1 and Orai1 [45]. In HEK293 cells that express a constitutively active SGK1, for instance, increased expression levels of STIM1 mRNA have been found [12]. In mast cells from mice lacking functional SGK1, STIM1 and Orai1 were downregulated [12].

In our experiments, preincubation with the SGK1-blocker EMD (50 μM) [47] one hour before and during Dex-treatment prevented upregulation of mRNA of STIM2 and TRPC channels 3 and 6. These results are in accordance with the findings of other groups linking GC-dependent SGK1-stimulation to the expression of ion channels and transporters important for excitation-contraction coupling. In HL-1 cardiomyocytes, Dex-incubation (500 nM, 24 h) increased expression of the sodium-proton exchanger NHE1 [48]. The effect was prevented in the presence of EMD (50 μM) and did not occur in SGK1-deficient cardiomyocytes.

In our experiments, SGK1-inhibition by EMD was sufficient to completely prevent the SOCE-increase by Dex (Fig 3C). It is conceivable that changes in SOC channel expression may result in altered intracellular Ca cycling. STIM1 overexpression has been shown to increase Ca transient amplitude and SR Ca content adult rat cardiomyocytes [38,49,50]. In accordance, STIM1 knockdown has been shown to reduced SR Ca content in neonatal cardiomyocytes [50]. On the other hand, STIM1-transgenic mice showed an increased SOCE but unaltered SR Ca load, which was attributed to increased diastolic SR Ca leak that may counterbalance increased SOCE-dependent filling of the SR [49].

STIM1 location is not strictly restricted to the SR membrane, since approximately 10% of STIM1 are found in the plasma membrane [51–53]. STIM has been shown to interfere with non-selective cation channels of the TRPC protein family [13,54]. The TRPC channel family consists of 7 members, of which TRPC channels 1, 3–6 have also been shown to be directly or indirectly activated by STIM1 [13,54]. TRPC channels (except for TRPC channel 2) are expressed in human, mouse and rat cardiomyocytes [55,56]. TRPC3 and 6 have been shown to be involved in the development of cardiac hypertrophy in rat [57], mouse [58,59], and human [59] cardiomyocytes but may also contribute to Ca entry, which is in accordance with our results. The Dex-dependent increased TRPC3 and 6 mRNA expression may partly contribute to the observed increased Ca transient amplitude and contractility.

A caveat for the potential application of GC stimulation to increase SOCE and inotropy could be its interference with the cellular stress response leading to disturbed Ca handling and arrhythmias [60,61]. It was shown that GC stimulation results in increased ER stress, measured as reduced methylation of FK binding protein 51 (FKBP51) [60]. Importantly, increased ER stress has been linked to increased Ca spark activity and atrial fibrillation via increased phosphorylation and activation of CaMKIIδ in human, rabbit and mouse atrial tissue [62,63]. Moreover, direct stimulation of SOCE by high doses of 2-aminoethoxydiphenyl borate (2-APB; 20 μM) has been shown to induce arrhythmias and spontaneous contractions in an Orai-dependent fashion in rat atrial and ventricular myocytes [64]. The precise mechanisms for these pro-arrhythmogenic effects certainly warrant further investigation.

## Summary/Conclusion

Dex-pretreatment increased mRNA expression of store-operated Ca channels, which is accompanied by increased SOCE, Ca transient amplitude and fractional shortening. These changes were prevented in the presence of SOCE blockers SKF and BTB2. On the other hand, Ca transient kinetics, a measure of CICR (time to peak, [20]) and SERCA function (decay time, [20]) were unaltered suggesting that Dex, at least after 24 h exposure, preferentially stimulates SOCE, which differentiates the positive inotropic effects from other pharmacological pathways (i.e. β-adrenergic pathway). Given its slow kinetics, SOCE appears to influence cellular Ca cycling by an integrated response across multiple cardiac cycles but not on a beat-to-beat basis. Our data add to the growing body of evidence that SOCE, although not relevant for basal contractile function, may be important as a short-term protective response to cardiac stress.

## Supporting information

S1 Table Oligonucleotide sequences of the inner and outer PCR primers. (DOCX)

S2 Table Relative mRNAβ-actin-expression of SOCE channels (a.u.). (DOCX)

S1 Fig Additional analysis of Ca handling in isolated FURA-2 loaded ventricular myocytes. (A) Analysis of mean data for fractional SR release (calculated as Ca transient amplitude normalized to Caffeine transient amplitude). Exposure of ventricular myocytes to Dex (24h) did not affect fractional SR Ca release. (B) Mean data for rate constant k derived from single exponential fits of the caffeine-induced (10 mM) Ca transient decay, which can be used as a measure of NCX function. There was no significant difference in k between the experimental groups. n = 6–9 animals for each group. (C-F) Correlation (Pearson r) analysis for Ca transient amplitude and caffeine-transient amplitude of isolated ventricular myocytes exposed to either vehicle (C), Dex (D), Dex and BTP2 (E) or Dex and SKF (F) within individual ventricular myocytes (n = 8–20 cells for each group). Linear regression lines and coefficients of determination (r^2^) are also shown for each plot. There was a strong correlation between Ca transient amplitude and caffeine-transient amplitude for each experimental group.
(TIF)

## Abbreviations

2-APB: 2-Aminoethoxydiphenyl borate
a.u.: Arbitrary units
BDM: 2,3-butanedionmonoxime
BSA: Bovine serum albumine
BW: Bodyweight
Ca: Calcium
CICR: Calcium-induced calcium release
Dex: Dexamethasone
EMD: EMD638683
GC: Glucocorticoid
i.p.: Intraperitoneally
LTCC: L-type calcium channels
LVEDP: Left ventricular end diastolic pressure
MEM: Minimal essential medium
NCX: Sodium-calcium exchanger
Orai: ORAI calcium release-activated calcium modulator 1
RyR: Ryanodine receptor
SEM: Standard error of the mean
SERCA: Sarco-/endoplasmatic reticulum calcium-Transporting ATPase
SGK1: Serum and glucocorticoid-regulated kinase 1
SKF: SKF-96365
SOCC: Store-operated Ca channel
SOCE: Store-operated calcium entry
SR: Sarco-/endoplasmic reticulum
STIM: Stromal interaction molecule
TRPC: Transient receptor potential cation channel
