# Supplementary material for: Glucocorticoid stimulation increases cardiac contractility by SGK1-dependent SOCE-activation in rat cardiac myocytes
Source: PLoS One. 2019 Sep 9;14(9):e0222341. doi: 10.1371/journal.pone.0222341 (PMC6733454; doi:10.1371/journal.pone.0222341)
Supplement: S1 Table — (DOCX) [file pone.0222341.s001.docx]

**S1 Table. Oligonucleotide sequences of the inner and outer PCR primers.**

| **mRNA** | **Accession number** | **Orientation** | **Primer sequence (5'-3')** | **Amplicon** |
| --- | --- | --- | --- | --- |
| **rTRPC1** | NM_053558 | forward | CGACACCTTCCACTCGTTCA | 254bp |
|  |  | reverse | CGACACCTTCCACTCGTTCA |  |
| **rTRPC3** | NM_021771 | forward | GCTTGTGTTCAACGCCTCAG | 173bp |
|  |  | reverse | ACAGCTCCTTGCACTCAGAC |  |
| **rTRPC4** | NM_001083115 | forward | ACGCCATCAGGAAAGAGGTG | 134bp |
|  |  | reverse | GATAGGCGTGATGTCTGGGG |  |
| **rTRPC6** | NM_053559 | forward | TGGCAAGTCCAGCATACCTG | 182bp |
|  |  | reverse | CTCCGTGTTTCTGCAGAGGT |  |
| **rSTIM1** | NM_001108496 | forward | TTGTCCATGCAGTCCCCCA | 242bp |
|  |  | reverse | AGAGATCCTGGATGGACCCC |  |
| **rSTIM2** | NM_001105750 | forward | CGACATGTTTGCGAGAACGG | 166bp |
|  |  | reverse | CCCGCAATAGGGTAAGGTGG |  |
| **rORAI1** | NM_001013982 | forward | ACGTCCACAACCTCAACTCC | 362bp |
|  |  | reverse | ACTGTCGGTCCGTCTTATGG |  |
| **rORAI2** | NM_001170403 | forward | CACCTATTTGCCCTGCTCAT | 386bp |
|  |  | reverse | AGCTTGTGCAGTTCCTCGAT |  |
| **rORAI3** | NM_001014024 | forward | GCGGCTACCTCGACCTTATG | 246bp |
|  |  | reverse | CCATGAGTGCAAACAGGTGC |  |
| **rActb** | NM_031144 | forward | CCACCATGTACCCAGGCATT | 189bp |
|  |  | reverse | CGGACTCATCGTACTCCTGC |  |

TRPC: transient receptor potential canonical channel, STIM: stromal interaction molecule, ORAI: ORAI calcium release-activated calcium modulator, Actb: beta-actin.
