# Supplementary material for: Glucocorticoid stimulation increases cardiac contractility by SGK1-dependent SOCE-activation in rat cardiac myocytes
Source: PLoS One. 2019 Sep 9;14(9):e0222341. doi: 10.1371/journal.pone.0222341 (PMC6733454; doi:10.1371/journal.pone.0222341)
Supplement: S2 Table — (DOCX) [file pone.0222341.s002.docx]

**S2 Table. Relative mRNA/β-actin-expression of SOCE channels (a.u.)**

| **Channel** | **Vehicle** | **N** | **DEX** | **N** | **DEX+EMD** | **N** |
| --- | --- | --- | --- | --- | --- | --- |
| **TRPC1** | 0.7884±0.1149 | 18 | 1.091±0.1877 | 13 | 0.8530±0.06263 | 7 |
| **TRPC3** | 1.297±0.2874 | 15 | 2.955±0.8891 | 7 | 0.2440±0.1302 | 5 |
| **TRPC4** | 0.5072±0.1065 | 17 | 1.897±0.5855 | 13 | 1.338±0.1060 | 6 |
| **TRPC6** | 0.3856±0.08760 | 16 | 1.419±0.3870 | 12 | 0.2551±0.07961 | 7 |
| **STIM1** | 0.5056±0.06306 | 18 | 0.8308±0.1156 | 12 | 1.045±0.1069 | 8 |
| **STIM2** | 0.8433±0.1024 | 18 | 1.671±0.2684 | 13 | 1.091±0.1326 | 8 |
| **ORAI1** | 4.875±1.258 | 14 | 1.619±0.3481 | 11 | 3.987±1.150 | 7 |
| **ORAI2** | 0.9914±0.4671 | 11 | 0.2194±0.09874 | 11 | 0.1304±0.03727 | 6 |
| **ORAI3** | 0.5035±0.08239 | 18 | 0.8317±0.1419 | 13 | 0.6587±0.08660 | 7 |

TRPC: transient receptor potential canonical channel, STIM: stromal interaction molecule, ORAI: ORAI calcium release-activated calcium modulator, Actb: beta-actin.
