# Supplementary figures and images for: Glucocorticoid stimulation increases cardiac contractility by SGK1-dependent SOCE-activation in rat cardiac myocytes
Source: PLoS One. 2019 Sep 9;14(9):e0222341. doi: 10.1371/journal.pone.0222341 (PMC6733454; doi:10.1371/journal.pone.0222341)

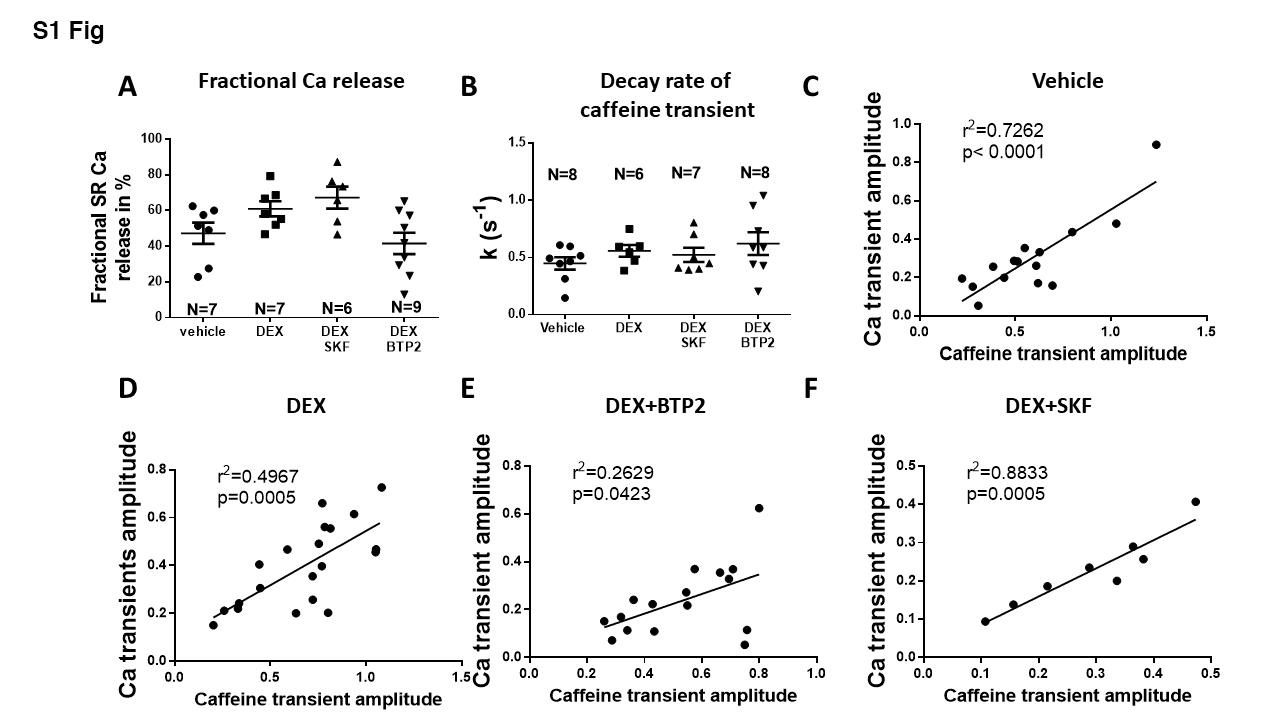

Supplement: S1 Fig — (A) Analysis of mean data for fractional SR release (calculated as Ca transient amplitude normalized to Caffeine transient amplitude). Exposure of ventricular myocytes to Dex (24h) did not affect fractional SR Ca release. (B) Mean data for rate constant k derived from single exponential fits of the caffeine-induced (10 mM) Ca transient decay, which can be used as a measure of NCX function. There was no significant difference in k between the experimental groups. n = 6–9 animals for each group. (C-F) Correlation (Pearson r) analysis for Ca transient amplitude and caffeine-transient amplitude of isolated ventricular myocytes exposed to either vehicle (C), Dex (D), Dex and BTP2 (E) or Dex and SKF (F) within individual ventricular myocytes (n = 8–20 cells for each group). Linear regression lines and coefficients of determination (r2) are also shown for each plot. There was a strong correlation between Ca transient amplitude and caffeine-transient amplitude for each experimental group. (TIF) [file pone.0222341.s003.tif]
